# Combination Treatment with Sublethal Ionizing Radiation and the Proteasome Inhibitor, Bortezomib, Enhances Death-Receptor Mediated Apoptosis and Anti-Tumor Immune Attack

**DOI:** 10.3390/ijms161226238

**Published:** 2015-12-21

**Authors:** Ercan Cacan, Alexander M. Spring, Anita Kumari, Susanna F. Greer, Charlie Garnett-Benson

**Affiliations:** 1 Department of Molecular Biology and Genetics, Gaziosmanpasa University, 60250 Tokat, Turkey; ercan.cacan@gop.edu.tr; 2 Department of Biology, Georgia State University, 161 Jesse Hill Jr. Dr, Atlanta, GA 30303, USA; aspring1@gsu.edu (A.M.S.); akumari1@student.gsu.edu (A.K.); 3 Department of Clinical Research and Immunology, American Cancer Society, 250 Williams St, Atlanta, GA 30303, USA; susanna.greer@cancer.org

**Keywords:** radiation, proteasome, death receptors, anti-tumor immunity

## Abstract

Sub-lethal doses of radiation can modulate gene expression, making tumor cells more susceptible to T-cell-mediated immune attack. Proteasome inhibitors demonstrate broad anti-tumor activity in clinical and pre-clinical cancer models. Here, we use a combination treatment of proteasome inhibition and irradiation to further induce immunomodulation of tumor cells that could enhance tumor-specific immune responses. We investigate the effects of the 26S proteasome inhibitor, bortezomib, alone or in combination with radiotherapy, on the expression of immunogenic genes in normal colon and colorectal cancer cell lines. We examined cells for changes in the expression of several death receptors (DR4, DR5 and Fas) commonly used by T cells for killing of target cells. Our results indicate that the combination treatment resulted in increased cell surface expression of death receptors by increasing their transcript levels. The combination treatment further increases the sensitivity of carcinoma cells to apoptosis through FAS and TRAIL receptors but does not change the sensitivity of normal non-malignant epithelial cells. Furthermore, the combination treatment significantly enhances tumor cell killing by tumor specific CD8^+^ T cells. This study suggests that combining radiotherapy and proteasome inhibition may simultaneously enhance tumor immunogenicity and the induction of antitumor immunity by enhancing tumor-specific T-cell activity.

## 1. Introduction

Colorectal cancer (CRC) is the third most common cancer type and the five year survival rate is less than 30% for advanced colorectal cancer [1]. Immunotherapies offer a promising modality for the treatment of advanced cancers because the immune system is systemic and thus able to attack metastatic disease [2,3]. Tumor-specific cytotoxic T lymphocytes (CTLs) and activated natural killer (NK) cells play particularly important roles in cancer cell killing and are the basis of many immunotherapies [4,5].

One way to improve tumor cell killing by CTLs or NK cells is to enhance expression of death receptors on tumor cells. DR4 (TRAIL-R1), DR5 (TRAIL-R2) and Fas (CD95/Apo-1) are members of the tumor necrosis factor receptor superfamily (TNFRSF), and ligation of death receptors by binding with cognate death ligands from anti-tumor immune cells induces apoptotic signals into tumor cells [6]. Fas is the complementary receptor for Fas-ligand (FasL) and this interaction plays an important role in triggering apoptosis. During cancer progression, the interaction between Fas and FasL is largely impaired due to suppression of Fas expression on tumor cells [7,8,9]. DR4 and DR5 are receptors for the tumor necrosis factors-related apoptosis-inducing ligand (TRAIL) and they are also essential for driving apoptosis in many types of tumor cells [10]. TRAIL is highly expressed in NK cells and CD8^+^ T cells [11], and it is part of a natural mechanism to kill tumor cells by the immune system and selectively induces apoptosis in cancer cells with less toxicity towards healthy/non-cancerous cells [12]. However, tumor cells often down-regulate cell surface expression of death receptors in order to avoid elimination by immune cells [13,14]. Thus, enhancing the expression of these death receptors on cancer cells could increase tumor cell sensitivity to CTL-mediated killing.

We have shown that sub-lethal doses of radiation can modulate gene expression, making tumor cells more susceptible to immune responses including enhancing T-cell-mediated immune attack [15,16,17]. While radiation is a useful tool to make tumor cells more susceptible to immune cells [18], effective immunotherapy approaches need to be developed for the treatment of multiple advanced cancer types. The 26S proteasome is a large protein complex formed by 19S regulatory and 20S core subcomponents, and found in the nucleus and cytoplasm of eukaryotic cells [19]. The 26S proteasome is the main non-lysosomal protein degradation machinery and inhibition of the 26S alters protein turnover and impacts cellular homeostasis [20]. Inhibition of the 26S also alters expression of numerous target genes at the transcriptional level by increasing the stability of transcription factors and/or epigenetic modifiers [21,22]. Bortezomib is the first FDA approved 26S proteasome inhibitor and is currently used for the treatment of multiple myeloma and mantle cell lymphoma [23]. Bortezomib specifically inhibits the chymotrypsin-like activity of the 26S [24].

It has been reported that bortezomib sensitizes melanoma tumors to dendritic cell-activated immune responses [25] and to TRAIL-mediated apoptosis [26,27]. Recent clinical trials demonstrate the feasibility of using bortezomib concurrently with carboplatin/paclitaxel and radiation in non-small cell lung cancer [28], and the combination of histone deacetylase and proteasome inhibitors was shown to enhance CD8^+^ T cell responses in a preclinical cervical cancer model [29]. However, it remains unclear if the combination of radiation and proteasome inhibition alters immune responses against tumors. Here we hypothesize that a combination treatment of sub-lethal radiation and bortezomib will increase expression of death receptors in CRC cells, which will make these cancer cells more susceptible to death-receptor mediated cell killing, and will enhance the CTL-mediated anti-tumor immune attack. Our specific goal is to increase expression of death receptors in CRC cells by a combination of sub-lethal radiation and inhibition of the 26S proteasome to enhance CTL-mediated tumor killing. Our data demonstrate that a combination of 26S proteasome inhibition and sub-lethal radiation significantly increases the sensitivity of carcinoma cells, but not normal non-malignant epithelial cells, to apoptosis. Combination treatment increases cell surface expression of multiple death receptors by increasing transcriptional activation of each gene. Our studies suggest that combining radiotherapy and proteasome inhibition may simultaneously enhance tumor immunogenicity and the induction of antitumor immunity by enhancing tumor-specific T-cell activity.

## 2. Results

### 2.1. Effects of Combination Treatment on Colorectal Cancer Cell Viability

**Figure 1:**
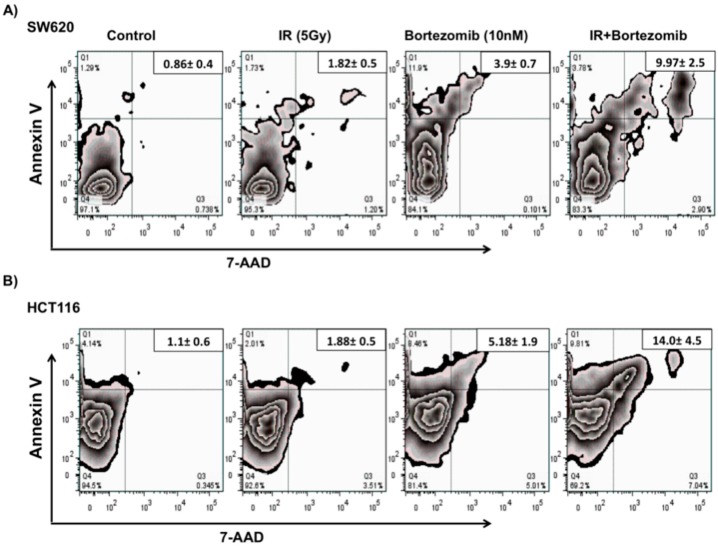
Tumor cells remain viable after a combination treatment of proteasome inhibitor and sub-lethal irradiation. Tumor cells were mock-irradiated (0 Gy) or irradiated with 5 Gy and cultured for 24 h. Following incubation, mock-irradiated or irradiated cells were treated with 10 nM bortezomib and incubated for an additional 24 h. An Annexin V-PE Apoptosis Detection Kit I (BD PharMingen, San Diego, CA, USA) was used for staining; results were quantified by Flow cytometry analysis and were analyzed using FlowJo software (FlowJo LLC, Ashland, OR, USA). Experiment was repeated three times with similar results. The relative increase of dead tumor cells in (**A**) SW620 (**B**) and HTC116 colorectal cancer cells.

To investigate the effects of the 26S proteasome inhibitor bortezomib, in combination with radiotherapy, on tumor cell death we used two well characterized colorectal cancer cell lines (SW620 and HCT116). The tumor cells were mock-irradiated (0 Gy) or irradiated with 5 Gy and were re-cultured for 24 h. Following incubation, mock-irradiated or irradiated cells were treated with 10 nM bortezomib and were incubated for an additional 24 h. Cell viability was detected based on Annexin V and 7 AAD staining (Figure 1). Flow cytometric analysis was used to distinguish between populations of live (Annexin V and 7 AAD double negative), apoptotic (Annexin V single positive) and dead (Annexin V and 7 AAD double positive) cells.

Greater than 90% of the cells remained viable after treatment with IR alone, as previously reported, and greater than 80% remained viable following bortezomib treatment. Interestingly, the combination treatment significantly (*p* < 0.005) increased the population of cells that are positive for both Annexin V-PE and 7-AAD (late apoptotic and dead cells). The observed values for dead cells went from 0.86% (untreated) to 9.97% (combination treated) of SW620 cells (Figure 1A), and from 1.1% (untreated) to 14.0% (combination treated) of HTC116 cells (Figure 1B). However, approximately 80% of SW620 and 70% of HCT116 cells remained viable even after combination treatment with both treatments. Our data demonstrate that most tumor cells remain viable after a combination treatment of sub-lethal irradiation and proteasome inhibitor, however the combination treatment enhances tumor cell death as compared to control or individual treatments.

### 2.2. Combined Treatment Does Not Inhibit the Initial DNA Repair Response

With the observed increase in cellular apoptosis after combined treatment, single cell gel electrophoresis (Comet assays) was used to evaluate whether the combined treatment negatively impacts the DNA damage response. Comet assays allow for a direct visualization of the extent of DNA damage: the greater the damage, the larger the “tail” of the comet [30]. As cells repair DNA damage, the extent of the comet tail will diminish. Thus, a comparison of results at equal time-points will give insight into differences in the DNA damage repair response following different treatment conditions. To probe for bortezomib’s potential interference in the DNA repair process, cells were pretreated with bortezomib prior to low dose radiation treatment and then assayed at early time-points in order to evaluate any changes in the initial DNA damage repair response. SW620 cells were either untreated or treated with 10 nM bortezomib and allowed to incubate for 24 h. After incubation, the cells were harvested and either mock-irradiated (0 Gy) or irradiated with 10 Gy and then immediately placed on ice or allowed to incubate at room temperature for 20 min followed by ice for 10 min prior to preparation for comet assays under alkaline conditions. The latter incubation conditions allow for approximately 50% DNA damage repair to occur in untreated irradiated cells.

**Figure 2:**
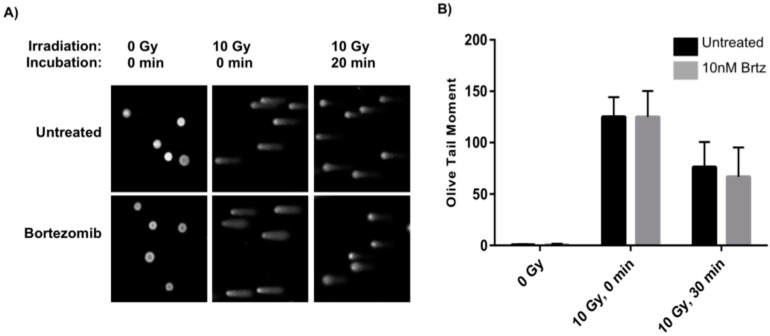
Initial DNA damage response is not inhibited by 26S proteasome inhibition. (**A**) SW620 cells were untreated or treated with 10 nM bortezomib, incubated for 24 h, mock-irradiated (0 Gy) or irradiated with 10 Gy, and either immediately placed on ice and prepared for comet assays or incubated for 20 min at room temperature followed by 10 min on ice and prepared for comet assays; (**B**) Olive tail moments for non-irradiated, irradiated with 10 Gy with no incubation, and irradiated with 10 Gy with incubation were compared for untreated (black) and bortezomib treated (gray) cells. Data for irradiated cells are the average of two independent experiments with error bars denoting standard deviation.

As anticipated, non-irradiated cells (both untreated and treated with 10 nM bortezomib) have a near zero Olive tail moment due to a lack of induced DNA damage. Irradiated cells that were not incubated at room temperature exhibit the maximum tail moment due to a lack of a DNA damage repair response; for theses assays, there was no difference in the Olive moments between bortezomib treated cells versus untreated cells (Figure 2; 0 Gy & 0 min). Cells that were allowed to incubate for 20 min at room temperature and 10 min on ice allowed for approximately 50% DNA repair as seen in the Olive moment; again for these assays, there was no difference in the Olive moment between the bortezomib treated cells versus the untreated cells. (Note, when cells were allowed to incubate at 37 °C post irradiation, the DNA damage repair was rapid and comet tails were not large enough for analysis (data not shown); in contrast, room temperature incubation slowed the repair process in order to garner insight into the any impacts on the DNA repair process). All results shown are representative of duplicate experiments; more than 75 measurements were taken for each condition. These data establish that the observed slight increase in apoptosis is not a result of impaired response to initial DNA damage. DNA damage repair occurs rapidly, within the first 2 h of damage [31]. As such, colorectal cancer cells treated with low dose irradiation followed by bortezomib treatment resulted in cells with no DNA damage after 24 h incubation (data not shown).

### 2.3. Combination Treatment Further Enhances Transcript Expression of DR4, DR5 and Fas over Radiation or Inhibition of the 26S Proteasome Alone Treated Carcinoma Cells

**Figure 3:**
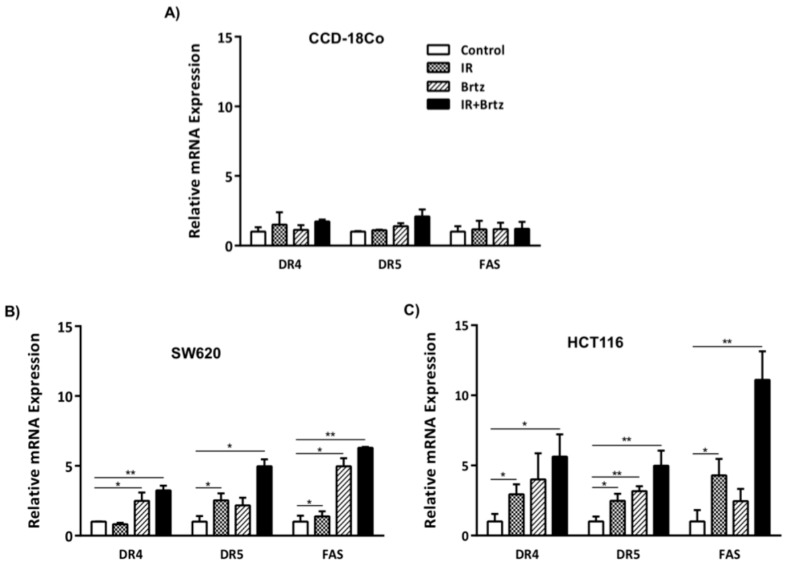
Inhibition of the 26S proteasome enhances transcript expression of death receptors with combination of radiation in tumor cells. Following the treatments, adherent cells were harvested, RNA was extracted and cDNA was generated. Data was quantified using qRT-PCR with primers and probes specific for DR4, DR5 or Fas coding regions and the obtained data were normalized to housekeeping gene HPRT1 expression. Graphed data shows the average of three independent experiments, with error bars denoting SEM. * *p* < 0.05, ** *p* < 0.005. Relative mRNA expression of DR4, DR5 and Fas in (**A**) CCD-18Co, (**B**) SW620 (**C**) and HTC116 cells.

The role of proteasome inhibition in the expression of death receptors in response to radiation has not been investigated. We began our investigation by treating cells with either 5 Gy radiation, 10 nM bortezomib, or the combination in order to detect altered transcript expression of death receptors. We further included a normal human cell line into our investigation to compare alterations in expression of death receptors between non-malignant and malignant human cells. The non-malignant human cell line, CCD-18Co, and two malignant carcinoma cell lines, SW620 and HCT116, were irradiated for 24 h and were then treated with bortezomib for an additional 24 h. DR4, DR5 and Fas mRNA expression was then quantified by qRT-PCR. No alteration in the transcript expression of death receptors was observed in the normal colon CCD-18Co cell line (Figure 3A) following neither the individual treatments nor the combination. In contrast, bortezomib upregulated the transcript expression of DR4, DR5 and Fas by 2.5-, 2- and 5-fold in SW620 cells (Figure 3B). While radiation only increased DR5 transcript expression by 2-fold in SW620 cells, the combination treatment of radiation and bortezomib significantly increased the transcript expression of DR5 by 5.9-fold. DR4 and Fas expression were increased by 4.6- and 7.2-fold after combination treatment.

To determine if increase in the expression of these genes is a common mechanism in carcinoma cells, we evaluated a second human CRC cell line, HCT116. 5 Gy radiation increased transcript expression of death receptors up to 5-fold and bortezomib treatment upregulated the transcript expression of DR4, DR5 and Fas by 4.5-, 3.6- and 2.4-fold (Figure 3C). The combination treatment of radiation and bortezomib significantly increased the transcript expression of DR4, DR5 and Fas by 6.8-, 5.2- and 12.1-fold (black bars). Overall, HCT116 cells were more responsive to these treatments than SW620 cells. However, the highest quantities of death receptor mRNA for both cells lines were detected following treatment of CRC cells with combination of radiation and bortezomib.

### 2.4. Combination Treatment with Bortezomib and Radiation Up-Regulates Cell Surface Protein Expression of Death Receptors in Tumor Cells

**Figure 4:**
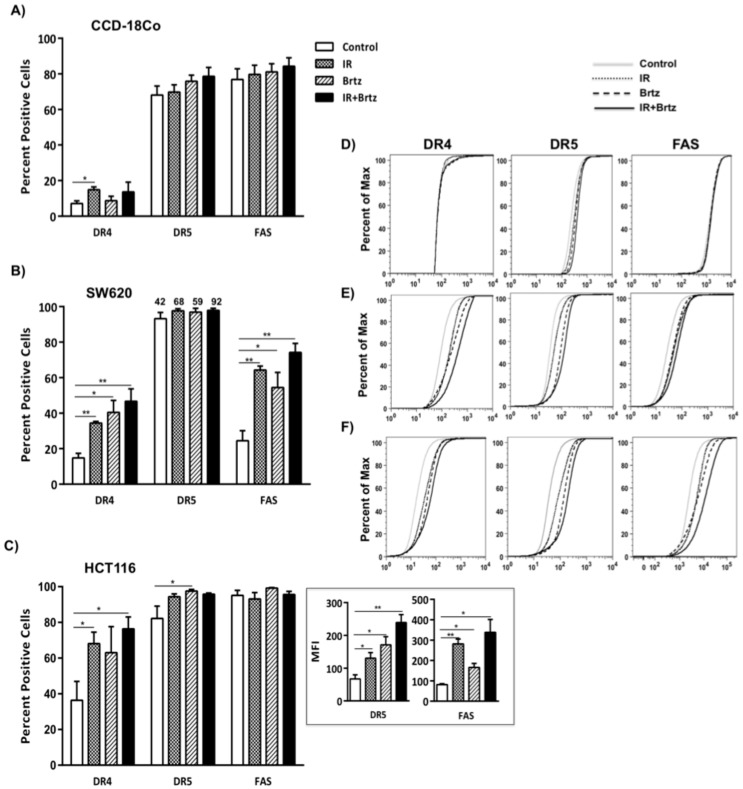
A combination treatment of sub-lethal dose of radiation and proteasome inhibition increases cell surface expression of death receptors. Following the treatments, cells were harvested and stained with PE-labeled antibody to human DR4, Fas or APC-labeled DR5. Cell surface protein expression was evaluated by flow cytometry. Isotype control stained cells were set to 5% positive. Graph represent average of three independent experiments, with error bars denoting SEM * *p* < 0.05, ** *p* < 0.005. Cells surface expression of DR4, DR5 and Fas in (**A**) CCD-18Co, (**B**) SW620 (**C**) and HTC116 cells. Representative cumulative distribution function (CDF) plots of DR4, DR5 and Fas expression in (**D**) CCD18Co, (**E**) SW620 and (**F**) HCT116 cells.

Next, to determine if combination treatment of proteasome inhibition and sub-lethal radiation could synergize to alter protein expression of DR4, DR5 and Fas, we investigated cell surface expression of these death receptors following treatment of non-malignant and malignant cells. Normal CCD-18Co colon fibroblast cells expressed low level of DR4 without treatment and the surface expression slightly increased with IR treatment but was never detected in greater than 20% of the normal cells. Untreated CCD-18Co colon cells expressed high levels of DR5 and Fas on the surface, but neither radiation nor bortezomib treatment significantly altered DR5 or Fas surface expression on CCD-18Co cells (Figure 4A). In contrast to CCD-18 Co cells, both individual treatments and the combination treatment significantly increased cell surface protein expression of DR4, DR5 and Fas in both colorectal carcinoma cell lines (Figure 4B,C). High levels of DR5 surface expression on untreated SW620 and HCT116, and Fas surface expression on HCT116 cells, make it difficult to see changes in the frequency of cells expressing these receptors. However, changes in the median fluorescence intensity (MFI) values reveal a substantial change in the expression (density) levels of both DR5 and Fas following radiation, bortezomib or combination treatment (Figure 4C; insets). Representative FACS plots show that the treatments modulate expression of death receptors only in malignant tumor cells, not in normal colon cells and the better increase in the expression of these death receptors was observed with the combination treatment in SW620 and HCT116 malignant cells (Figure 4D–F). Consistent with mRNA expression data, the combination treatment had a considerable impact on protein expression of death receptors in both tumor cells as compared to untreated cells. In contrast, irradiation and inhibition of the 26S proteasome did not impact the near normal cell line. Overall, these data suggest that the combination treatment had little impact on the expression of death receptors in normal colon cell line but significant effect on expression of these proteins in colorectal cancer cells.

### 2.5. Proteasome Inhibition Can Further Increase Radiation-Induced Sensitivity to Killing of CRC Cells by CD8^+^ T Cells

**Figure 5:**
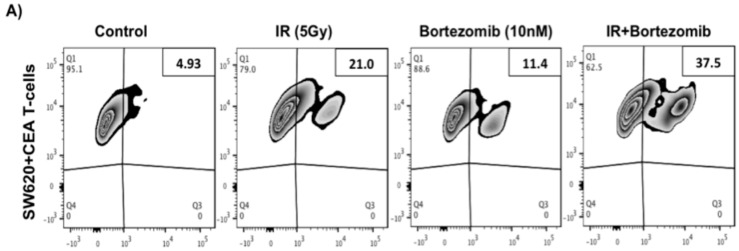

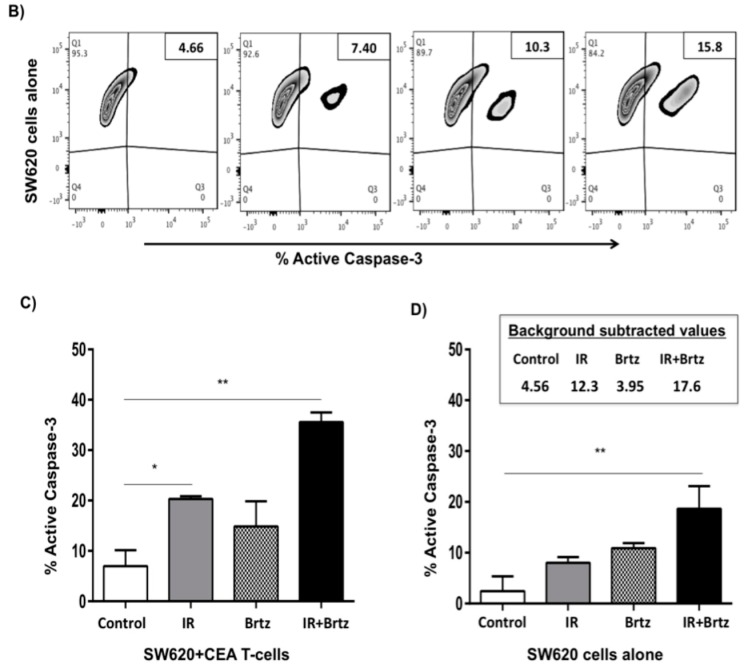
Combination of sub-lethal irradiation and bortezomib or irradiation alone enhances the killing of CRC mediated by CTLs. (**A**) SW620 cells were treated with 5 Gy, bortezomib or combination of radiation and bortezomib. Cells were harvested and co-incubated with human CEA specific CTLs (E:T ratio 10:1) for 3.5 h at 37 °C in a 96 well plate. The frequency of tumor cells expressing active caspase-3 was determined by flow cytometry and data was analyzed by flowjo software; (**B**) As a negative control, SW620 cells were treated and incubated under similar condition as described above in the absence of CTLs; (**C**,**D**) Bar graph showing the average of two additional replicate experiments. Error bars represent the SEM. * indicates *p* value <0.05. ** indicate *p* value <0.005. CEA = Carcinoembryonic antigen and E:T, effector cell to target cell ratio.

To test if the combination treatment enhances colorectal cancer cell sensitivity to CTLs, SW620 cells were irradiated (5 Gy) or treated with combination irradiation plus bortezomib prior to incubation with carcinoembryonic antigen (CEA)-specific CD8^+^ T cells. The level of active caspase-3 was evaluated in tumor cells by flow cytometry after co-culturing with CEA specific CTLs as a measure of cells undergoing caspase-dependent cell death. Tumor cells displayed significantly increased levels of caspase-3 after irradiation (21.0%) and combination treatment with irradiation and bortezomib (37.5%) following incubation with tumor-specific T cells (Figure 5A). The combination treatment resulted in higher killing than radiation treatment alone. In the absence of CEA specific T cells the caspase-3 expressions was low after each of the different treatments (control—4.66%, 5 Gy—7.4%, combination—15.8%; Figure 5B) similar to the low levels of cell death seen by Annexin-V and 7AAD viability analysis (Figure 1). Background subtracted values from the average of three independent experiments show that radiation (12.3%) and the combination (17.6%) treatment greatly increase tumor-specific T cell activity against tumor cells (Figure 5C,D). Thus, these data suggest that colorectal carcinoma cells treated with combination irradiation and bortezomib are even more sensitive to killing mediated by tumor antigen specific T cells than tumor cells treated with radiation alone.

### 2.6. Proteasome Inhibition Can Further Increase Radiation-Induced Sensitivity to Killing through FasL and TRAIL Receptors

**Figure 6:**
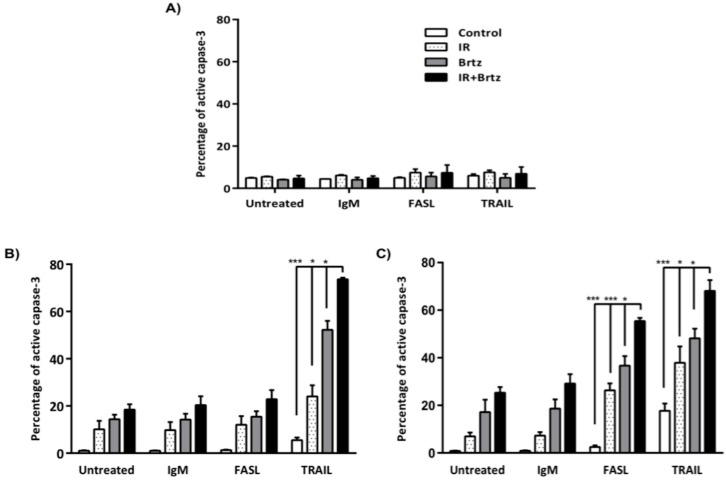
Inhibition of the 26S proteasome and sub-lethal irradiation can enhance sensitivity to killing through FAS and TRAIL receptors in colorectal carcinoma cells, but not in CCD-18Co cells. Tumor cells were mock-irradiated (0 Gy) or irradiated with 5 Gy and cultured for 24 h. Following incubation, mock-irradiated or irradiated cells were treated with 10 nM bortezomib and incubated for an additional 24 h. The tumor cells were then incubated for 3 h with agonistic anti-Fas antibody (FASL) or recombinant TRAIL protein. Control cells were incubated with IgM isotype control antibody. Cells were subsequently fixed and permeabilized before being stained for intracellular active caspase-3 with a PE-labeled monoclonal antibody. The level of activated caspase-3 was evaluated by flow cytometry. Isotype control stained cells were analyzed for each treatment group individually and set to 5% positive. Graph shows average of three independent experiments, with error bars denoting SEM * *p* < 0.05, *** *p* < 0.0005. Percentage of active caspase-3 in (**A**) CCD-18Co, (**B**) SW620, (**C**) and HTC116 cells inhibition sensitizes tumor cells to Fas or TRAIL mediated killing possibly through enhancing cell surface expression of death receptors, which sensitizes tumor cells to killing by their ligands.

To investigate if enhanced expression of DR4, DR5 and Fas on colorectal cancer cells by radiation and bortezomib treatment is functional, cells were mock-irradiated (0 Gy) or were irradiated with 5 Gy and re-cultured for 24 h. Following incubation, mock-irradiated or irradiated cells were treated with 10 nM bortezomib and were incubated for an additional 24 h. Cells were then incubated for 3 h with of agonistic anti-Fas antibody or recombinant TRAIL protein. The level of activated caspase-3 was used to determine the percentage of apoptotic cells by flow cytometry. Bortezomib and sub-lethal irradiation did not sensitize near normal colon CCD-18Co cells to killing by anti-Fas or recombinant TRAIL protein (Figure 6A). SW620 cells are known to be insensitive to Fas-mediated cell death [15,32], and were used as negative control. As expected, neither radiation nor bortezomib treatment sensitized these cells to killing by the anti-Fas antibody, but we observed a significant cell death with TRAIL treatment (Figure 6B).

Conversely, irradiation alone or in combination with bortezomib significantly sensitized HCT116 cells to killing by both anti-Fas and TRAIL treatments (Figure 6C). IR, bortezomib or combination treatments lead to some background killing of HCT116 cells as we observed in SW620 cells. We also found a significant increase with combination treatment as compare to irradiation or bortezomib treatment alone following anti-Fas and TRAIL treatments, and we observed a significant cell death with treatments in HCT116 cells. Interestingly, bortezomib treatment alone also shows high TRAIL sensitivity, but the combination treatment further sensitizes SW620 and HCT116 cells to TRAIL mediated cell death. These data suggest that the combination treatment is more impactful on sensitization of colorectal cancer cells to Fas and TRAIL mediated cell death. These data further suggest that sub-lethal radiation or proteasome.

## 3. Discussion

Proteasome inhibitor, bortezomib, stimulates multiple signaling cascades, primarily the NF-kB pathway, to induce apoptosis [33,34,35]. The combination treatment of bortezomib with other agents has been widely studied [36]. Most of these studies focus on additive or synergistic effect of a combination treatment to directly induce apoptosis [37,38,39]. Here we focused on the effect of bortezomib and radiation on gene expression that mediates immune mediated apoptosis. Our work significantly contributes to the cancer immunotherapy field by using the combination of sub-lethal radiation and proteasome inhibitor in controlling the expression of death receptors on tumor cells for sensitivity to cytolysis by CTLs. In this study, we show that the combination treatment of bortezomib and sub-lethal radiation significantly increases the cell surface expression of multiple death receptors by increasing their transcriptional abundance and surface expression.

We started to investigate the effects of the 26S proteasome inhibitor, bortezomib, alone or in combination with radiotherapy, on direct induction of cell death in two colorectal cancer cell lines. We found that the level of radiation utilized in these experiments is sub-lethal and the bortezomib concentration is very lowly lethal in colorectal cancer cell lines. Despite an increase in cell death with the combination treatment, over 80% of tumor cells remain viable (Figure 1). We also evaluated the possibility of an impairment in the initial DNA damage response as a potential mechanism for the increase in apoptosis. No difference in the extent of DNA damage was observed after the addition of bortezomib to radiation (Figure 2) indicating that the DNA damage response is not inhibited by the combination treatment and therefore not likely to be the cause of the increased apoptosis.

It has been shown that ionizing radiation effects proteasome structures [40] but the dynamics of the induction of proteasome inhibitor in irradiated cells is unclear, and the role of proteasome inhibition in expression of death receptors in response to radiation has not been investigated. Treatments significantly increased the transcript expression of DR4, DR5 and Fas in colorectal tumor cells (Figure 3). Interestingly, we did not see any alteration in the transcript expression of death receptor in normal colon CCD-18Co cell line. Consistent with mRNA data, individual treatments or the combination treatment significantly increase cell surface protein expression of DR4, DR5 and Fas in both colorectal carcinoma cell lines (Figure 4).

One of the most effective cancer immunotherapy strategies is to generate tumor-associated antigen (TAA) specific CTLs that are capable of killing tumor cells [41,42,43]. TAAs are derived from normal cellular proteins that have been mutated or are overexpressed by tumor cells [44]. For example, carcinoembryonic antigen (CEA) peptide is highly expressed in several cancer types, including CRC, and the immune system is not tolerant to these tumor-derived antigens [45]. The generation of CEA specific CTL responses in human has been under investigation in clinical trials for several cancer types [46,47,48]. Thus, enhancing tumor cell recognition by CTLs could increase tumor killing rate by TAA specific CTLs. Our results demonstrate that bortezomib and sub-lethal irradiation dramatically enhanced the percentage of SW620 colorectal cancer cells killed by CEA-specific T cells and the combination treatment further increased the percentage of apoptotic cells upon interaction with T cells (Figure 5). These data suggest that sub-lethal radiation or proteasome inhibition sensitize tumor cells to death-receptor mediated apoptosis, possibly through upregulation of death receptors, which sensitizes tumor cells to CTL-mediated killing.

Activation of caspase-3 is known as the endpoint of the caspase cascade that facilitates apoptosis [49]. Thus, we measured the frequency of cells with active caspase-3 to identify the number of apoptotic cells following treatment with anti-Fas or recombinant TRAIL protein to test if enhanced expression of death receptors by irradiation and bortezomib treatment would in fact increase sensitivity to killing through FasL or TRAIL receptors in tumor cells. Our data indicate that bortezomib and sub-lethal irradiation did not sensitize normal colon CCD-18Co cells to killing by anti-Fas (Figure 6A). However, irradiation itself or with combination of bortezomib significantly sensitized HCT116 cells to killing by anti-Fas (Figure 6C). Consistent with previous data, none of the treatments sensitized SW620 cells to killing by anti-Fas [15,32] (Figure 6B). SW620 cells have acquired genetic defects in apoptotic pathways and thus are resistant to FAS mediated apoptosis, which could be a potential mechanism of how some colon cancer cells escape the immune system. Importantly, these cells were still killed better by T cells suggesting that they can be rendered sensitive to attack when modulated by radiation and bortezomib. TRAIL selectively induces apoptosis in tumor cells by binding both DR4 and DR5 death receptors [50]. Our results show that bortezomib and sub-lethal irradiation did not sensitize CCD-18Co cells to killing by TRAIL (Figure 6A). However, the treatments significantly enhanced the percentage of active caspase-3 in both colorectal cancer cell lines (Figure 6B,C). Despite having defects in FAS-mediated apoptosis, SW620 cells were sensitized to TRAIL-mediated apoptosis following radiation, bortezomib and the combination treatment.

Consistent with previous studies, bortezomib treatment did not alter expression of death receptors in normal colon cells [51], which suggest that bortezomib has tumor selectivity. It is unclear why normal cells show less response to bortezomib treatment, but this could be because tumor cells require more protein synthesis which increases their dependency on proteasomal degradation. Furthermore, we didn’t see much change in FAS and TRAIL induced apoptosis in normal cells following bortezomib or radiation treatment (Figure 6A). Conversely, tumor cells were much more sensitive than normal cells to proteasome inhibition and radiation treatment. Differences in sensitivity to TRAIL also do not correlate obviously with variable p53 status or tumor grade between the cell lines [52,53,54]. Thus, it could be due to a loss of checkpoint mechanisms in cancer cells; however further investigation needs to be done to clarify the issue.

Our findings suggest that a combination treatment of radiation and bortezomib can be used for the treatment of colorectal cancer by inducing increased sensitivity to tumor specific immune responses. Having sufficient expression of death receptors on tumor cells may enhance the ability of tumor-specific T-cell activity and sensitivity to tumor cells. Radiation has been commonly used for the treatment of several cancers [55,56] and proteasome inhibitors demonstrate broad anti-tumor activity in clinical and pre-clinical cancer models [36,57]. Combination of radiation and proteasome inhibition maybe usefully applied in combination with immunotherapy to enhance T cell reactivity against tumors. In the last decade, many studies of immunotherapy for the treatment of malignant cancers have brought new strategies and approaches for improving the prognosis of cancer. Thus, our findings further contribute to our understanding of how clinically approved agents may be used to enhance immunotherapy strategies for the treatment of advanced colorectal cancer.

## 4. Experimental Section

### 4.1. Reagents and Cell Lines

Bortezomib were purchased from LC Laboratories (Woburn, MA, USA). Colorectal tumor cell line HCT116 cells were generously provided from the Laboratory of Tumor Immunology and Biology, NCI, NIH. Human colorectal carcinoma cell line SW620 and near normal CCD-18Co cells were purchased from ATCC. All cells were cultured in media designated by ATCC for propagation and maintenance. Cells were incubated at 37 °C incubator with 5% CO_2_ and tested to ensure absence of Mycoplasma.

### 4.2. Irradiation

Tumor and normal cells were irradiated by using a RS-2000 biological X-ray irradiator (Rad source technology, Suwanee, GA, USA). Cells were irradiated at a dose rate of 2 Gy/min for 2.5 min by setting irradiator voltage and current at 160 kV and 25 mA. During irradiation, the cells were maintained in recommended media and kept on ice. Following irradiation, the culture media was replaced with the fresh media.

### 4.3. Apoptosis Assay

Apoptosis of tumor cells was assessed using the Annexin V-PE Apoptosis Detection Kit I Tumor cells were mock-irradiated (0 Gy) or irradiated with 5 Gy and re-cultured for 24 h. Following incubation, mock-irradiated or irradiated cells were treated with 10 nM bortezomib and incubated for an additional 24 h. The tumor cells were briefly trypsinized and harvested. The cells were then washed with cold PBS twice and resuspended in Annexin V binding buffer at a concentration of 1 x 10^6^ cells/mL. Cells were then transferred to 5 mL culture tubes containing 5 μL of Annexin V-PE and/or 5 μL of 7-Aminoactinomycin D (7-AAD). The samples were gently mixed and were incubated for 20 min at room temperature. Following the addition of 400 μL of Annexin V binding buffer to each tube, samples were analyzed and quantified by flow cytometry and resulting data were analyzed using FlowJo software. Viable cells were negative for both annexin V-PE and 7-AAD; early apoptotic cells were positive for annexin V-PE and negative for 7-AAD, whereas late apoptotic and dead cells were positive for both annexin V-PE and 7-AAD labeling.

### 4.4. Comet Assay

The extent of DNA damage and resulting DNA damage repair was assessed using single cell gel electrophoresis (Comet assay) under alkaline conditions. Briefly, SW620 cells were plated and cultured for 24 h, then treated or not treated with bortezomib and incubated for an additional 24 h. The tumor cells were rinsed with PBS to remove dead cells, briefly trypsinized, harvested, and gently resuspended in fresh media. Tumor cells were then mock-irradiated (0 Gy) or irradiated with 10 Gy and immediately placed on ice or allowed to incubate at room temperature for 20 min and then placed on ice. Approximately 1 × 10^4^ cells were gently mixed with previously melted 0.5% low-melting agarose in PBS at 37 °C, applied to a slide previously coated with 1% normal-melting agarose, allowed to solidify in a cold box, and then immediately placed in lysis buffer (2.5 M NaCl, 100 mM EDTA, 10 mM Tris, 0.02% Triton, pH 10). Following lysis, the slides were placed in an alkaline solution (300 mM NaOH, 1 mM EDTA) for 30 min and subjected to electrophoresis in alkaline solution for 30 min (33 V, 300 mA). Following electrophoresis, slides were washed 4 times with cold H_2_O, DNA was precipitated with cold 100% ethanol for 5 min, and slides were allowed to dry at room temperature overnight. The resulting gels were stained using Sybr Green I and visualized using an LSM 700 scanning confocal microscope. Comet images were analyzed with the OpenComet algorithm (v 1.3) [58]. Late apoptotic cells present a different profile in the comet assay due to the degradation of genomic DNA (a characteristic “hedgehog” shape with a small head and a large “fan like” tail); these profiles were not included in the assay. The Olive tail moment was used for analysis: Olive Tail moment = (Tail Mean — Head Mean) × % DNA in Tail/100. Statistical analysis was performed using GraphPad (GraphPad Software, Inc., La Jolla, CA, USA).

### 4.5. RNA Expression and Quantitative Real-Time PCR

mRNA was isolated using QIAzol RNA extraction reagent (Qiagen) as described in Cacan *et al*. [59]. Briefly, cells were lysed in QIAzol and agitated on a 3D rotator for 5 min. Two hundreds µL of chloroform was added and was incubated for 3 min at room temperature. Samples were centrifuged and the aqueous phase was transferred to an eppendorf tube. Five hundreds µL of isopropanol was added and was incubated for 10 min at room temperature. Following centrifugation, pellets were washed with 75% cold ethanol, centrifuged and resuspended in RNAse free water. RNA was quantified and cDNA was generated from 1 μg of total extracted RNA using an Omniscript Reverse Transcription Kit (Qiagen, Valencia, CA, USA). Following cDNA synthesis, quantitative real-time polymerase chain reaction was performed using TaqMan Universal PCR Master Mix (Qiagen) and specific primers and probes targeting gene of interest (Applied Biosystems; Fas/CD95; Hs00163653_m1, DR4/TNFRSF10A; Hs00269492_m1, DR5/TNFRSF10B; Hs00366278_m1 and HPRT1; Hs99999909) according to manufacturer’s protocol. Transcript expression was assessed using an ABI prism 7900HT Real-Time PCR System (Applied Biosystems, Carlsbad, CA, USA). Reactions were normalized against HPRT1 expression and calculations were performed using standard curves generated.

### 4.6. Cell Surface Staining and Flow Cytometry Analysis

Cell surface staining of tumor and normal cells were performed using the following primary labeled antibodies; Fas-PE, DR4-PE, DR5-APC and the appropriate isotype matched controls (BioLegend. San Diego, CA, USA). Surface staining was performed in cell staining buffer for 45 min on ice. Stained cells were acquired on a BD Fortessa flow cytometer. Dead cells were excluded from the analysis based on scatter profile. Isotype control staining was less than 5% for all samples analyzed.

### 4.7. CTL Killing Assay

Peripheral blood mononuclear cells (PBMCs) from HLA-A2^+^ donors were purchased from Hemacare (Van Nuys, CA, USA) for generation of antigen specific CTLs. PBMCs were cultured in AIM-V media (Life Technologies, Carlsbad, CA, USA) for 2 h to allow them to adhere to the culture flask. Non-adherent cells were removed for lymphocyte isolation. Adherent cells were cultured for a week in the presence of 100 ng/mL of human granulocyte-macrophage colony stimulating factor (GM-CSF) and 20 ng/mL of IL-4 (Miltenyi Biotec, Inc. Auburn, CA, USA) to induce dendritic cell differentiation. On day five, 500 ng/mL of CD40L (Millipore) were added to mature the dendritic cells (DC). On day seven, DCs were harvested and 1 × 10^5^ of DCs were plated in a 12-well plate and then were pulsed with 40 μg/mL of HLA-A^2^ binding CEA peptide (YLSGANLNL) for 4 h in 37 °C. CEA loaded DCs were irradiated with 50 Gy to inhibit DCs proliferation and processing of new antigens. CD8^+^ T cells were isolated from non-adherent PBMCs using immunomagnetic beads (Miltenyi Biotec Inc., Auburn, CA, USA), as described by the manufacturer. Isolated CD8^+^ T cells were co-cultured with peptide loaded DCs in the presence of 10 ng/mL of IL-7 and 30 U/mL of IL-2 (Millipore, Temecula, CA, USA) to promote T cells viability and clonal expansion. IL-7 and IL-2 were refreshed on the third day. T cells were re-stimulated every 7-days with freshly pulsed DCs and peptide as described above. After three in vitro stimulations T cells were isolated over ficoll and used for caspase-dependent killing assays. SW620 cells were treated with IR (5 Gy), bortezomib or combination of irradiation and bortezomib. After 48 h irradiation and 24 h bortezomib treatment, the tumor cells were harvested and co-cultured with CEA specific CTLs (E:T ratio 10:1) at 37 °C for 3.5 h. After co-incubation, tumor cells were harvested and stained with EpCAM (Epithelial cell adhesion molecule; Miltenyi Biotech, San Diego, CA, USA) followed by intracellular staining with active caspase-3 (BD PharMingen, San Diego, CA, USA).

### 4.8. Functional Death Receptor Assay

Cells were mock-irradiated (0 Gy) or irradiated with 5 Gy and re-cultured for 24 h. Following incubation, mock-irradiated or irradiated cells were treated with 10 nM bortezomib and incubated for an additional 24 h. The cells were harvested and counted. Cells were then incubated for 3 h with varying concentrations of agonistic anti-Fas antibody, clone CH11 (MBL, Watertown, MA, USA) or recombinant TRAIL protein (Millipore, Billerica, MA, USA). Control cells were incubated with IgM isotype control antibody (BD Biosciences, San Diego, CA, USA). Cells were subsequently fixed and permeabilized before being stained for intracellular active caspase-3 with a PE-labeled monoclonal antibody (BD Biosciences San Diego, CA, USA). Stained cells were acquired on a BD Fortessa flow cytometer (BD PharMingen, San Diego, CA, USA). The level of activated caspase-3 was quantified by flow cytometry, as described above.

### 4.9. Statistics

Results were statistically evaluated using Student paired *t* test. The *p* values <0.05 are indicated by one asterisk (*). The *p* values <0.005 are indicated by two asterisks (**). The *p* values <0.0005 are indicated by three asterisks (***).

## 5. Conclusions

This study suggests that the combination of radiation and the proteasome inhibitor, bortezomib, may simultaneously enhance tumor immunogenicity and the induction of antitumor immunity by enhancing tumor-specific T-cell activity and sensitivity to death receptor mediated apoptosis. Findings in the manuscript indicate that combined treatment of bortezomib and radiation can be used as a potential therapeutic regimen for the treatment of advanced colorectal cancer with limited toxicity to normal non-malignant cells.
